# DOT1L and H3K79 Methylation in Transcription and Genomic Stability

**DOI:** 10.3390/biom8010011

**Published:** 2018-02-27

**Authors:** Katherine Wood, Michael Tellier, Shona Murphy

**Affiliations:** 1 Department of Biochemistry, University of Oxford, Oxford OX1 3RE, UK; katherine.wood-3@postgrad.manchester.ac.uk; 2 School of Biological Sciences, University of Manchester, Manchester M13 9PL, UK; 3 Sir William Dunn School of Pathology, University of Oxford, Oxford OX1 3RE, UK

**Keywords:** Dot1, DOT1L, H3K79me, transcription, genome stability, RNA polymerase II

## Abstract

The organization of eukaryotic genomes into chromatin provides challenges for the cell to accomplish basic cellular functions, such as transcription, DNA replication and repair of DNA damage. Accordingly, a range of proteins modify and/or read chromatin states to regulate access to chromosomal DNA. Yeast Dot1 and the mammalian homologue DOT1L are methyltransferases that can add up to three methyl groups to histone H3 lysine 79 (H3K79). H3K79 methylation is implicated in several processes, including transcription elongation by RNA polymerase II, the DNA damage response and cell cycle checkpoint activation. DOT1L is also an important drug target for treatment of mixed lineage leukemia (MLL)-rearranged leukemia where aberrant transcriptional activation is promoted by DOT1L mislocalisation. This review summarizes what is currently known about the role of Dot1/DOT1L and H3K79 methylation in transcription and genomic stability.

## 1. Introduction

Chromatin is the DNA–protein complex that organises genetic information within the nuclei of eukaryotic cells. The basic unit of chromatin is the nucleosome, which consists of 146 bp of DNA wrapped around an octamer of histone proteins; a H3/H4 tetramer and two H2A/H2B dimers [1,2,3,4]. Histones are subject to numerous reversible post-translational modifications, including acetylation, methylation, phosphorylation and ubiquitination. These covalent modifications influence chromatin structure and function directly, by altering the interactions between nucleosomes, and indirectly, by affecting the recruitment of non-histone effector proteins, such as transcription factors, chromatin remodellers and DNA binding proteins to particular regions of chromatin, which then drives downstream processes [5].

One well-studied covalent histone modification is the methylation of lysine residue by histone lysine methyltransferases (KMTs) [6]. This includes mono-, di- and tri-methylation and some of the best-characterised substrates are histone H3 lysine 4 (H3K4), lysine 9 (H3K9), lysine 27 (H3K27), lysine 36 (H3K36) and lysine 79 (H3K79), and histone H4 lysine 20 (H4K20). These methyl marks can contribute to the regulation of transcription, frequently acting as landing platforms for the recruitment of effector proteins [7]. Histone lysine methylation is also associated with other diverse functions, including heterochromatin formation, X chromosome inactivation, DNA repair, cell fate determination and terminal differentiation [6]. Misregulation of histone lysine methylation is associated with several human cancers and other diseases [8,9].

The KMTs that have been characterised thus far can be divided into two general groups on the basis of their catalytic domains. One group contains an evolutionarily-conserved catalytic SET (Su(var)3-9, Enhancer of Zeste and Trithorax) domain [10]. The other class does not contain a SET domain and consists of yeast Dot1 (disruptor of telomeric silencing-1; also known as Kmt4) [11] and its homologs in other organisms, which include the mammalian homologue DOT1L [12,13,14]. This second group contains a catalytic methylase domain related to that of class I methyltransferases, such as DNA methyltransferases and the protein arginine methyltransferase PRMT1 [15,16,17]. Dot1 and its homologs are involved in numerous processes, including transcriptional regulation, cell cycle progression and the DNA damage response [18].

The aim of this review is to discuss the role of Dot1/DOT1L in transcription and genome integrity.

## 2. Dot1/DOT1L Activity

Dot1/DOT1L (DOT1-Like) catalyse mono-, di- and tri-methylation of histone H3 lysine 79 in a non-processive manner [17,19] using S-adenosylmethionine (SAM) as a cofactor. These are the only known H3K79 methyltransferases, as knockout of their genes in yeast, flies, mice and humans leads to complete loss of H3K79 methylation [13,20,21].

Analysis of DOT1L activity has shown that it preferentially acts on H3K79 in the context of chromatin and is not active on free histones or recombinant H3 [12], suggesting that DOT1L might recognise other nucleosomal features. Unlike the majority of residues in histone H3 subject to covalent modification, which are within the N-terminal tail of the protein, K79 is located within a loop in the globular domain exposed on the surface of the nucleosome [2]. 

In yeast, monoubiquitination of histone H2B lysine 123 (H2BK123) is a prerequisite for H3K79 di- and trimethylation by Dot1, but not H3K79 monomethylation [22]. H2BK123 ubiquitination is established by Rad6 (ubiquitin-conjugating E2 enzyme) and Bre1 (ubiquitin E3 ligase). The Paf1 complex, associated with elongating RNA polymerase II (RNAPII), enhances the recruitment of Rad6 and Bre1 to chromatin, linking this modification to transcription elongation [23]. Deletion of Rad6 prevents both H3K4 and H3K79 methylation as well as H2BK123 ubiquitination, while mutagenesis of H2BK123 leads to the loss of both methylations [24,25,26]. In support of H2BK123 ubiquitination acting upstream of H3K79 methylation, Dot1 deletion does not affect H2BK123 ubiquitination [16]. Furthermore, in mammals, the deletion of MED23, which significantly reduces the H2Bub level in the gene body, decreases the H3K79me3 level but not the H3K4me3 signal [27]. H2BK123 and H3K79 are closely juxtaposed on the same solvent-exposed surface of the nucleosome [2], providing a structural basis for cross-talk between the two modifications. Several mechanisms have been proposed for how H2B ubiquitination regulates H3K79 methylation, including: an indirect interaction between Dot1 and H2BK123ub by an unknown protein; a direct interaction between Dot1 and H2BK123ub, which is supported by in vitro experiments showing that purified mononucleosomes containing ubiquitylated H2B were sufficient to directly stimulate DOT1L methyltransferase activity [28]; and structural changes to the nucleosome caused by monoubiquitination of H2BK123 that promote the methylation of H3K79 by Dot1. Some combination of these mechanisms may be operating, as they are not mutually exclusive. Similarly, in humans, monoubiquitination of histone H2B lysine 120 is required for efficient methylation of H3K79 by DOT1L [29].

Histone methylation, and in particular DOT1L-mediated H3K79 methylation, is a relatively stable modification. Studies using isotopic pulse labelling to measure the turnover rates for different histone modifications determined that the half-life of H3K79me1 and H3K79me2 in HeLa cells is 1.105 days and 3.609 days, respectively [30]. The half-lives of these histone methylation marks are much longer than the relatively short half-lives of modifications such as histone acetylation and phosphorylation, which are in the range of minutes to hours [30].

While the deposition of H3K79 methylation by DOT1L has been well-characterised, considerably less is known about the active removal of this modification. To date, H3K79 methylation is the only known histone lysine methylation without at least one corresponding histone demethylase [31,32,33,34,35]. However, there is considerable evidence suggesting that H3K79 methylation is reversible [36]. For example, the H3K79me2 level fluctuates with the cell cycle in *S. cerevisiae* and human cells [12,37], and a sudden loss of H3K79me2 has been observed during early development in flies and mice [21,38]. In addition, factors affecting the rate of cell division and replication-independent histone turnover influence the levels of H3K79 methylation and its genomic distribution. Identification of the elusive H3K79 demethylase would represent a considerable step towards fully understanding the role and regulation of DOT1L and its associated H3K79 methylation in biological processes.

## 3. H3K79 Methylation and Active Transcription

Genome-wide analysis of H3K79 methylation has demonstrated a high correlation between this modification and transcriptional activity. In *Saccharomyces cerevisiae*, approximately 10% of the genome is H3K79 hypomethylated while the remainder of the genome displays H3K79 methylation and is actively transcribed [13]. Additionally, it was shown that H3K79 methylation correlates with euchromatin at sites of active V(D)J (variable, diversity, and joining genes) recombination in mammalian cells, while hypomethylation is present at inactive loci [39]. Together, these suggest that H3K79 methylation is a marker of active euchromatin.

High-throughput technology allowing genome-wide mapping of specific histone modifications has allowed a more detailed insight into the role of H3K79 methylation in the regulation of transcription. In human CD4+ T cells, chromatin immunoprecipitation followed by high-throughput sequencing (ChIP-Seq) studies have demonstrated that H3K79me2/me3 are strongly correlated with gene activity [40], while in *Drosophila melanogaster* chromatin immunoprecipitation followed by DNA microarray (ChIP-chip) has also demonstrated a relationship between H3K79me2 and active transcription [41]. Mutations in the *Drosophila* Dot1 ortholog *grappa* are associated with mutant *Polycomb* and *Trithorax* phenotypes [21]. Polycomb and Trithorax are protein complexes involved in transcriptional regulation of numerous developmental genes [42], indicating that H3K79 methylation influences developmentally-regulated gene expression in metazoa. Steger et al. [43] demonstrated, using ChIP-chip, that H3K79 methylation is associated with RNAPII transcription in mouse 3T3 cells and that all H3K79 methylation marks are within the body of actively-transcribed genes, with the level of enrichment correlating to the level of gene expression. Genes where RNAPII has a high elongation rate also have higher H3K79me2 levels than more slowly-transcribed genes [44,45] and H3K79 methylation is enriched on the variant histone H3.3, which is associated with transcriptionally-active loci in mammals and *Drosophila* [46,47]. Furthermore, H3K79me2 is detected at expressed miRNA genes, as well as protein-coding genes [48]. Collectively, these genome-wide studies in yeast, fly, mouse, and humans indicate that H3K79 methylation is associated with active transcription.

DOT1L has been reported to directly interact with RNAPII phosphorylated on Ser2 and/or Ser5 of the C-terminal domain (CTD) of its largest subunit [49]. The CTD is an inherently unstructured yet highly evolutionarily-conserved domain, comprising between 26 (yeast) to 52 (human) tandem repeats of the consensus heptad YSPTSPS [50,51]. The CTD is subject to numerous reversible post-translational modifications on specific residues in both consensus and non-consensus repeats [51]. For example, the hyperphosphorylation of the CTD, principally on Ser5 and Ser2 of the repeats, corresponds to the promoter release of RNAPII and entry into productive transcription elongation, respectively [52]. The CTD serves as a flexible binding platform for numerous nuclear factors and changes in the modification patterns of the repeats as RNAPII transcribes a gene orchestrate the binding of different sets of proteins for specific functions at different stages of the transcription cycle. [51]. Thus, the interaction of DOT1L with phosphorylated Ser5 and/or Ser2 of the CTD could help recruit this enzyme to actively-transcribed genes.

Other proteins have been reported to interact with DOT1L. For example, Bat3 has been shown to interact with both DOT1L and H3 and is proposed to colocalise DOT1L and H3 to increase DOT1L enzymatic activity [53]. More recently, DOT1L has also been demonstrated to interact with the proto-oncogene c-Myc. C-Myc is essential for the presence of DOT1L and H3K79me2 at several genomic loci, suggesting that c-Myc targets the enzyme to these loci [54].

**Figure 1:**
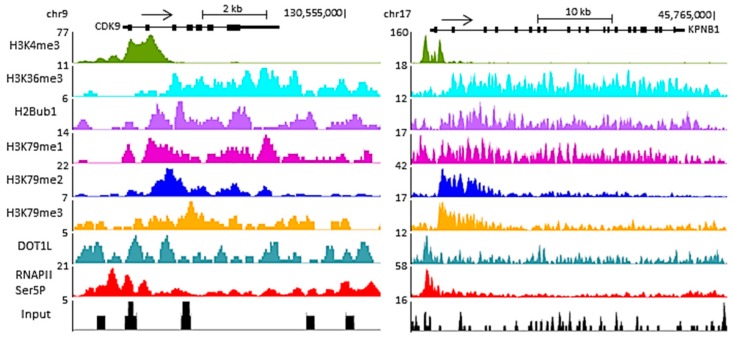
Representative genome browser track of read coverage profiles of RNA polymerase II (RNAPII) Ser5P (initiation complex), DOT1L and different histone marks (ubiquitination of histone 2B lysine 120 (H2BK120ub), histone 3 lysine 4 trimethylation (H3K4me3), histone 3 lysine 36 trimethylation (H3K36me3), histone 3 lysine 79 mon-, di- and tri-methylation (H3K79me1, H3K79me2 and H3K79me3, respectively)) on two active human protein-coding genes, *CDK9* and *KPNB1*, in the NCITT cell line (chromatin immunoprecipitation followed by high-throughput sequencing (ChIP-seq) data from [49,57]). Read coverage is presented on the left side of each ChIP-seq. The arrow above the gene represents the sense of transcription. All three H3K79 methylation states display a peak immediately downstream of the H3K4me3 peak but upstream of the H3K36me3 broad peak.

Interestingly, H3K79 methylation is not uniform within an expressed gene (Figure 1). H3K79me2 and H3K79me3 levels are highest immediately downstream of the transcription start site (TSS) and decrease gradually within the first intron [55,56]. H3K79me1 peaks in the same region as the di- and trimethylation but displays a broader distribution [43,56]. The peaks of H3K79 mono-, di- and trimethylation correspond to a region of transcription transition, located after the peak of H3K4me3 marking regions of transcription initiation but before the H3K36me3 mark observed in regions of transcription elongation (Figure 1). 

## 4. DOT1L in Transcriptional Elongation

Genome-wide investigations have demonstrated that H3K79 methylation is present in the coding regions of active genes [58], suggesting a role for DOT1L in transcription elongation. In support of this, Krogan et al. [59] showed that in yeast the Paf1 protein complex, which is associated with elongating RNAPII, regulates the H3K79 methyltransferase activity of Dot1. In mammalian cells, DOT1L has been purified in various RNAPII-associated transcription elongation complexes, summarized in Table 1. 

**Figure 2:**
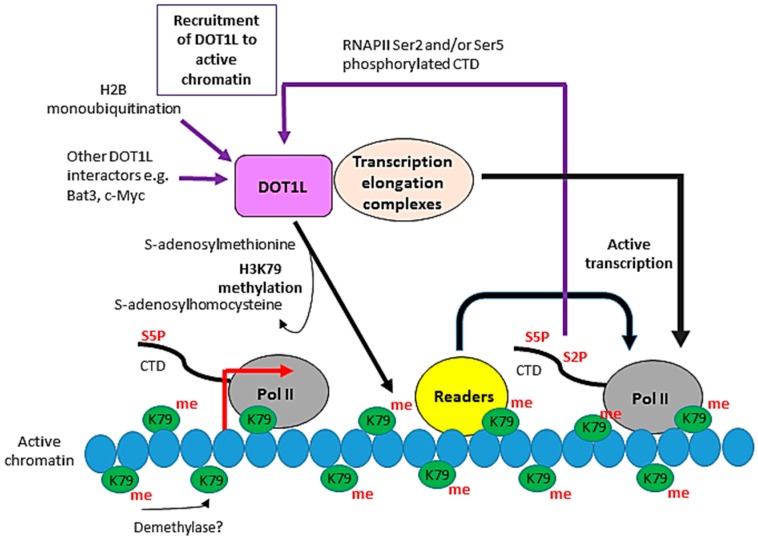
Function(s) of DOT1L in transcription. DOT1L is a histone H3 lysine 79 (H3K79) methylase which catalyses the mono-, di- and trimethylation of H3K79 in a non-processive manner using S-adenosylmethionine as a cofactor. No H3K79 demethylase has been identified, although there is evidence that H3K79 methylation is reversible. H3K79 methylation shows a high correlation with transcriptional activity. The requirement for H2B lysine ubiquitination for DOT1L activity and the interaction of DOT1L with RNA Pol II phosphorylated on serine 2 and/or serine 5 of its C-terminal domain (CTD) are potential mechanisms recruiting DOT1L to active genes. DOT1L is found in various eukaryotic transcription elongation complexes and its activity varies with the activity of members of these complexes and hence transcription elongation, supporting a role for DOT1L in the transcription of at least a subset of genes. H3K79 methylation likely functions in transcription via the recruitment of “readers” which act directly or indirectly to affect RNA Pol II activity.

The DOT1L-associated complex ENL-associated proteins (EAP) and the core EAP complex contain several transcription elongation factors and the positive transcription elongation factor b (P-TEFb). P-TEFb contains cyclin-dependent kinase 9 (CDK9) activity, which is required for the phosphorylation of Ser2 of the CTD of RNAPII, an event coincident with the transition of RNAPII from initiation to the productive elongation phase of transcription (Figure 2). The EAP complex thus contains both H3K79 methyltransferase activity and RNAPII CTD kinase activities. Crucially, knockdown of ENL, AF9 and AF10 reduces both H3K79me2 levels across the genome and global transcription elongation activity by RNAPII, indicating that these proteins regulate DOT1L [64]. The DOT1L-containing AF4-associated complex also contains P-TEFb. Overexpression of AF4, AF9, AF10 and ENL increases both P-TEFb-dependent transcription elongation and levels of H3K79 methylation [61]. Taken together, these support a function for DOT1L in transcription elongation by RNAPII.

More recently, DOT1L was found in *Drosophila* in a complex, DotCom, which contains members of the Wnt pathway and AF10, AF17 and AF9. Although P-TEFb is not present in this complex, DOT1L is necessary for expression of Wingless target genes, supporting a function in transcription [63]. However, another study performed in mouse intestinal epithelia found that DOT1L and H3K79me2 were not required for the expression of Wingless target genes, questioning the requirement of DOT1L for the Wnt pathway in mammals [67]. However, the purified super-elongation complex (SEC) and AEP (composed of AF4, ENL and P-TEFb) elongation complex (Table 1) contain several transcription elongation factors but lack DOT1L. Variations in the composition of different protein complexes may have resulted from differences in methods and purification procedures employed, although it is possible that several transcription elongation complexes exist and DOT1L is only associated with a subset of these. Indeed, the complex containing AF4/AFF4:AF9 has been found to be mutually exclusive with the complex containing DOT1L:AF9 [62]. The reason behind this is the binding of DOT1L and AF4 to the same intrinsically disordered domain of AF9/ENL [68]. Nonetheless, DOT1L clearly has a role in the transcription of at least a subset of genes, most likely by facilitating transcription elongation.

The mechanisms by which H3K79 methylation regulates transcription remain unclear. Crystallographic structures of recombinant nucleosomes with either unmethylated H3K79 or an H3K79me2 mimic have demonstrated that there are no significant differences in global nucleosomal architecture and only minor local conformational changes [69]. Thus, H3K79 methylation is likely to function indirectly via the recruitment of effector proteins. The PWWP (named after a conserved Pro-Trp-Trp-Pro motif) domain of hepatoma-derived growth factor 2 (HDGF2) binds H3K79me3 [70], while the Tudor domain of survival of motor neuron protein (SMN) [71] and the tandem Tudor domains of 53BP1 and fragile X mental retardation protein (FMRP) also bind H3K79me [72,73]. However, additional, as yet undiscovered readers could serve as a platform for the recruitment of proteins involved in transcription elongation. Alternatively, H3K79 methylation may inhibit the binding of repressors [74,75]. 

## 5. Dot1 and Telomeric Silencing in *Saccharomyces Cerevisiae*

**Figure 3:**
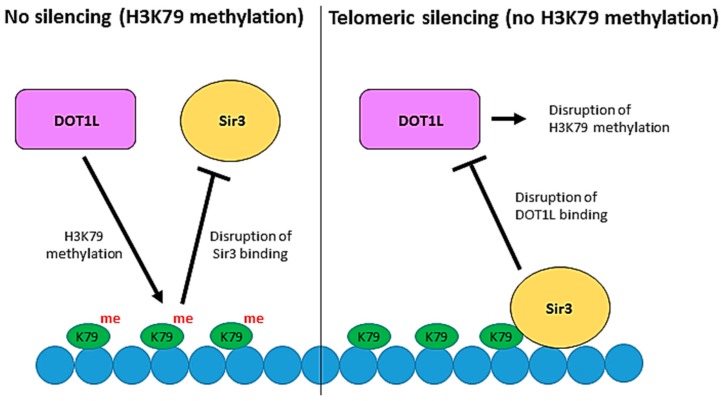
The role of Dot1 in telomeric silencing. Telomeric DNA silencing in *Saccharomyces cerevisiae* is established by the binding of the Sir proteins, including Sir3, to chromatin. Competition between H3K79 methylation by Dot1 and Sir protein binding regulates heterochromatin formation at telomeres. Methylation of H3K79 prevents the binding of Sir3 and therefore disrupts telomeric silencing. Conversely, binding of Sir3 to unmethylated H3K79 prevents the interaction of Dot1 with chromatin by outcompeting Dot1 for binding to a basic patch on histone H4 which is essential for Dot1 recruitment and H3K79 methylation.

Despite the firm evidence linking Dot1 to actively transcribed loci, Dot1 was originally implicated in the silencing of genes in the telomeres of yeast. Indeed, Dot1 was identified in a genetic screen as a protein that disrupts telomeric silencing when overexpressed in *Saccharomyces cerevisiae*. Telomeric and telomere-proximal DNA silencing is established via the recruitment and binding of Sir (silent information regulator) proteins [76]. Mutation of H3K79 or deletion of Dot1 compromises silencing at telomeric loci by disrupting Sir protein localisation. Chromatin immunoprecipitation (ChIP) analysis demonstrated that Dot1 overexpression and deletion both lead to mislocalisation of the Sir protein complex (Sir2, 3, 4) [13]. Cells overexpressing Dot1 display H3K79 methylation spreading into silent chromatin, suggesting that H3K79 methylation displaces Sir proteins from the silent regions of chromatin. In vitro and in vivo, Sir3 can bind to histone H3, but H3K79 methylation prevents the interaction and subsequently disrupts the spreading of heterochromatin [74]. Conversely, it has been shown that a basic patch on histone H4 is critical for Dot1 binding and H3K79 methylation and that Sir3 competes with Dot1 for the same site on H4 [74,77]. The correct balance of Sir protein binding and H3K79 methylation levels by Dot1 was therefore thought to be crucial for regulating heterochromatin formation at telomeres (Figure 3).

However, the effect of Dot1 inhibition or overexpression on heterochromatin formation of telomeres is probably a consequence of Dot1 functions during transcription. Indeed, Rossman et al. [78] showed, using a *URA3* telomere reporter assay, that the silencing defect in Dot1 mutants is rather due to an imbalance in ribonucleotide reductase and a *URA3* promoter at telomere VII-L rather than a need for Dot1 in general telomere silencing. Takahashi et al. [79] also demonstrated that the role of Dot1 in heterochromatin formation is telomere-specific. Nonetheless, the competition between Sir proteins and Dot1 may also regulate telomeric heterochromatin formation, although this may be less important that initially believed.

## 6. Dot1/DOT1L and the DNA Damage Response

Studies in both yeast and mammalian cells have demonstrated a clear link between Dot1/DOT1L, H3K79 methylation and the DNA damage response (DDR).

As mentioned, the tandem Tudor domain of the human DNA repair protein 53BP1 binds to H3K79me and is recruited to DNA double strand breaks (DSBs) [72]. Mutation of H3K79 or knockdown of DOT1L both suppress the recruitment of 53BP1 to DSBs. The yeast ortholog of 53BP1, Rad9, also contains a Tudor domain which interacts with H3K79me [80]. Since the levels of H3K79 methylation are unchanged upon DNA damage, it has been suggested that DSBs are responsible for structural changes in the chromatin, which lead to the exposure of H3K79 for recognition by 53BP1 [72]. However, more recently, it has been suggested that H4K20me2, and not H3K79me, is the main histone target for 53BP1 recruitment to DSBs in mammalian cells [81,82,83]. However, H3K79me may be important for 53BP1 recruitment when H4K20me levels are low or absent [53]. For example, when budding yeast, in which H4K20me is absent, are treated with ionising radiation (IR), which induces DSBs, in the G1 phase of the cell cycle, they typically undergo a G1 checkpoint delay. Dot1 mutants are defective in both G1 and intra-S checkpoints and progress through the cell cycle normally even after IR-induced DNA damage [80]. These checkpoint defects are also observed in mutants of Dot1 activity (such as by mutation of H3K79 or disruption of H2BK123 ubiquitination) following genotoxic stress [84,85,86]. Therefore, Rad9-dependent checkpoint activation following IR-mediated DNA damage in the G1 phase is dependent on Dot1. 

**Figure 4:**
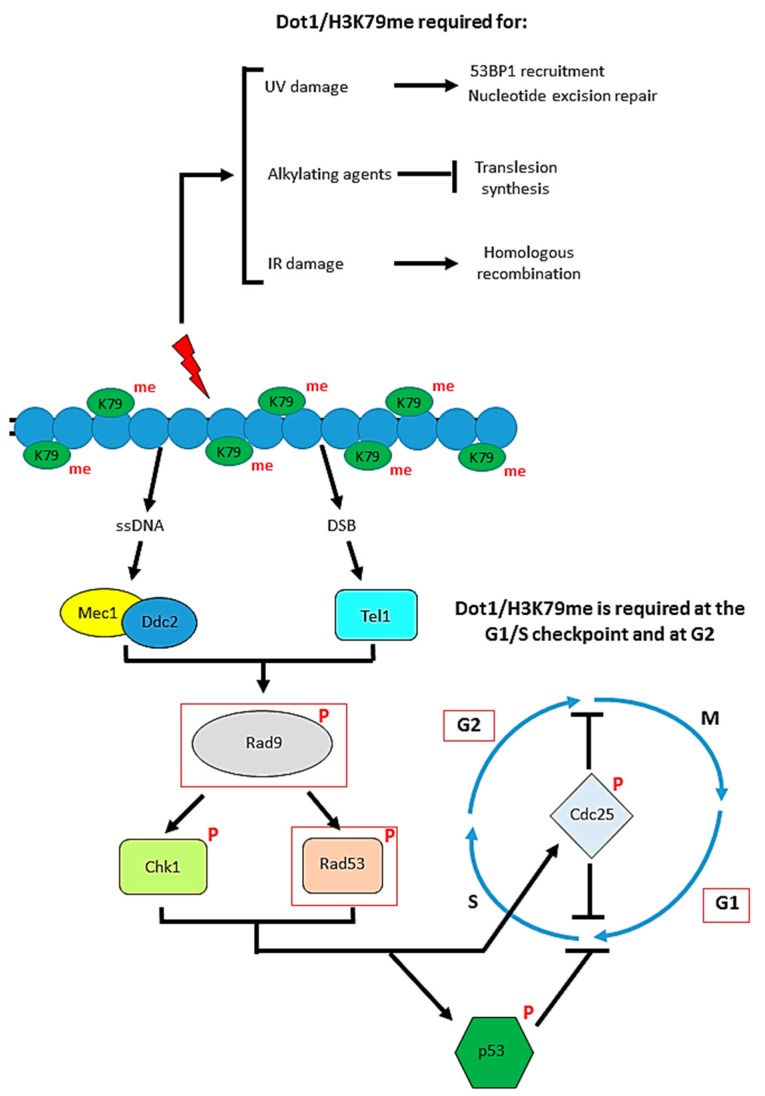
The role of Dot1 in the DNA damage response. The yeast proteins are shown, and the steps in which Dot1 has a positive effect are outlined in red. Several different DNA repair pathways can be used to respond to different types of DNA damage, and Dot1 is implicated in a number of these. Loss of H3K79me impairs ultraviolet (UV)-induced lesion repair pathways, leading to UV-hypersensitivity. Dot1 maintains genomic integrity by inhibiting the translesion synthesis (TLS) pathway in response to alkylating agents. Recruitment of Dot1 and subsequent recruitment of Rad9 are required for regulating the 5′–3′ resection step in homologous recombination (HR)-mediated repair of ionizing radiation (IR)-induced DNA double-strand breaks (DSBs). Additionally, H3K79 methylation is required for cohesin recruitment, key for maintaining chromosome structure and for efficient sister chromatid recombination (SCR) to repair DSBs Dot1 is also required for checkpoint function at both G1 and G2. Following recognition and binding of sensor proteins to DNA damage sites, mediator proteins including Rad9 are recruited and activated by phosphorylation. H3K79 methylation by Dot1 is essential for the recruitment of Rad9 and phosphorylation of the downstream transducer protein Rad53, allowing the cell to enter an arrest phenotype at G1/S or G2 upon DNA damage.

In addition to its G1 checkpoint role, it has been shown that Dot1 confers IR-resistance mediated by promoting homologous recombination (HR) repair of DSBs [85]. While Dot1 deletion mutants do not show a G2 arrest phenotype [85], Rad9 recruitment to sites of DNA damage and phosphorylation of Rad53, a downstream transducer protein (see Figure 4), following IR treatment still requires Dot1 [87]. 

Therefore, it is thought that Dot1-mediated H3K79me is required at two distinct stages of the Rad9-dependent DNA damage response: an early step corresponding to G1/S checkpoint activation, and, at a later G2 stage, DNA repair [88].

There are two main pathways for the repair of DSBs: non-homologous end-joining (NHEJ) and HR. An important initial step in HR is the resection of DSBs to produce 3′ single-stranded (ss)DNA tail intermediates. H379 methylation and Rad9 recruitment are key regulatory factors in this resection step, limiting the extent of ssDNA production. This is thought to prevent the activation of checkpoint proteins in response to accumulated ssDNA as part of DNA repair, hence contributing to a tightly controlled DNA damage response [89]. In addition, HR with the sister chromatid recombination (SCR) in mitosis is key for the accurate transmission of DNA. Proteins such as cohesin are essential for maintaining chromosome structure and efficient SCR, and not only does Dot1 have an essential role in the recruitment of Rad9 to DSBs for DNA resection, it also promotes the recruitment of cohesin for efficient SCR [90].

H3K79 methylation by Dot1 is also important in other forms of DNA repair. For example, adducts such as (6-4) photoproduct dimers and cyclopyrimidine dimers are caused by ultraviolet radiation (UV), and can be repaired by a number of different pathways, including nucleotide excision repair (NER) and recombination repair (RR). Dot1-dependent H3K79me is crucial in the repair of these UV-induced DNA lesions [84], as the loss of these histone marks results in UV hypersensitivity [89,91]. In addition to Dot1 function in the Rad9-regulated DNA damage checkpoint, a direct role of Dot1 in NER is supported by the similarity in the UV survival pattern of a *dot1Δ*, *rad1Δ* and *dot1Δ rad1Δ* yeast strain [91]. This indicates that Dot1 and Rad1, an enzyme of the NER creating a 5′ incision at the site of UV damage [92], act in the same pathway. The base excision repair (BER) pathway is involved in the repair of DNA damage caused by, for example, alkylating agents. When this pathway fails, translesion synthesis (TLS) is used to achieve cell cycle progression and hence survival, using error-prone DNA polymerases to bypass the lesions during DNA replication. It has been shown that Dot1 negatively regulates TLS, promoting genomic integrity following DNA damage [93,94,95]. These roles of Dot1 in the DNA damage response are summarized in Figure 4.

Similarly, studies in mammalian cells have demonstrated that knockdown of DOT1L increases sensitivity to IR and UV radiation [53,96,97]. However, whether this is via disruption of 53BP1 recruitment or through alternative mechanisms, such as affecting chromatin structure, which impacts RNAPII reactivation and transcriptional restart (as proposed by Oksenych et al. [97]), is still unclear. Nonetheless, the clear involvement of Dot1/DOT1L in the DNA damage response emphasizes the key role this enzyme plays in genomic integrity.

## 7. H3K79 Methylation and Leukemia

**Figure 5:**
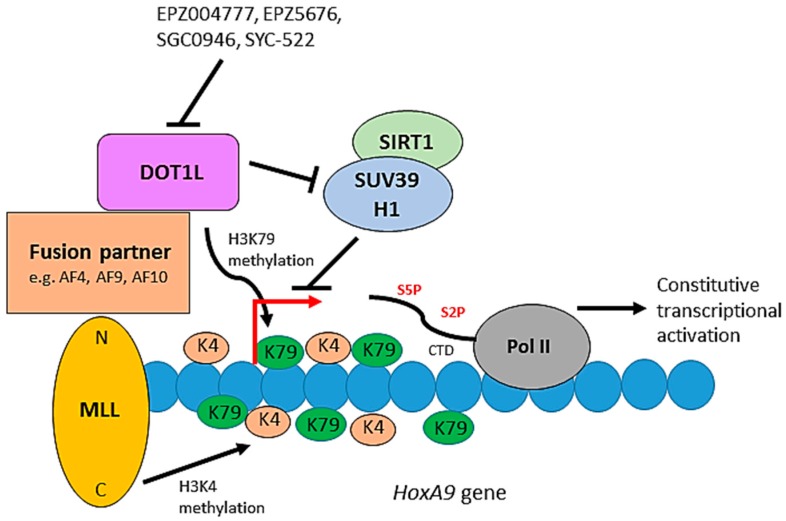
The role of DOT1L in leukemogenesis. The C-terminus of MLL is an H3K4 methyltransferase. Constitutive activation of a leukemic transcriptional program, including the *HoxA9* gene, occurs through mislocalisation of DOT1L and its associated proteins through the interaction of DOT1L with fusion partners of the MLL N-terminus. DOT1L-mediated H3K79 hypermethylation and inhibition of the SIRT1-SUV39H1 complex, which normally represses MLL fusion target genes, promote the constitutive activation of the target genes resulting in leukemic transformation. Small molecules can inhibit DOT1L methyltransferase activity blocking de novo H3K79 methylation and leukemogenic gene expression.

Aberrant transcriptional activation via H3K79 methylation by DOT1L has been implicated in the development of leukemias that derive from oncogenic chromosomal rearrangements of the *MLL* (mixed lineage leukemia) gene. As a result of chromosomal translocations, the N-terminus of MLL becomes fused in-frame to one of approximately 70 translocation partners [98]. A subset of these MLL fusion partners, including AF4, AF6, AF9, AF10 and ENL, cause aberrant recruitment of DOT1L, leading to H3K79 hypermethylation and constitutive transcriptional activation of genes required for leukemogenesis (Figure 5). For example, an MLL-AF10 fusion promotes DOT1L-mediated methylation of H3K79 at the *HoxA9* promoter, which contributes to upregulation of expression of *HoxA9* in acute myeloid leukemia. Overexpression of *HoxA9* and the transformation capability of MLL-AF10 are dependent on DOT1L enzymatic activity, while deletion of the *HoxA9* gene prevents transformation by MLL-AF10 [99]. Thus, although DOT1L is not genetically altered as such, the mislocalisation of its methyltransferase activity and the activation of a leukemic transcriptional program is a consequence of the chromosomal translocations observed in MLL patients. DOT1L is therefore involved in leukemogenesis, especially in leukemias mediated by MLL fusion partners such as AF4, AF9, AF10 and ENL, which account for two-thirds of all MLL-linked leukemias [100]. While the precise mechanism by which DOT1L contributes to the gene activation process requires further investigation, a recent study showed that DOT1L inhibits the recruitment of a repressive complex composed of SIRT1 and SUV39H1, a H3K9 methyltransferase, on MLL fusion target genes, thus maintaining an open chromatin state allowing gene expression [75]. 

Inhibition of DOT1L activity or disrupting the interaction between DOT1L and MLL fusion partners are potential therapeutic strategies for the treatment of MLL-fusion-related leukemias. EPZ004777 was identified as a small molecular inhibitor of DOT1L, acting by competing with the SAM cofactor required for DOT1L methyltransferase activity. This compound inhibits cellular H3K79 methylation, blocks leukemogenic gene expression and selectively kills cultured cells which contain MLL translocations, but has poor pharmacological properties [101]. A second-generation inhibitor, EPZ5676, is currently in clinical trials for the treatment of MLL-rearranged leukemias, and while the results look promising [102], the low bioavailability of the drug is a complicating factor [103]. Two other recent small molecules targeting DOT1L with high specificity have also been developed, SGC0946 and SYC-522 [101,104]. Further work towards a better understanding of the biology of DOT1L will help both to understand the effects of the currently-available inhibitors and to develop alternative strategies to target the DOT1L pathway for therapeutic applications.

## 8. Concluding Remarks

The highly-conserved enzyme Dot1/DOT1L methylates histone H3 lysine 79 and is important in many aspects of cell biology and genomic integrity, including transcriptional regulation and the DNA damage response. Furthermore, mammalian DOT1L is essential for embryogenesis, hematopoiesis and cardiac function [18]. The mislocalisation of DOT1L activity is strongly associated with leukaemias resulting from oncogenic chromosomal translocations involving the *MLL* gene. While targeting DOT1L is showing promise in terms of therapy for these diseases, the ongoing problems with current DOT1L inhibitors, such as low bioavailability and rapid clearance, mean that targeting DOT1L regulators or downstream effectors may become an attractive alternative way to modulate this pathway in disease. It is worth noting that DOT1L is extremely important in development [105] and a large proportion of MLL-linked leukaemia patients are infants [106], making alternative treatment options all the more important.

While genome-wide correlation studies have provided some insights over recent years, much remains unknown about the downstream effects of H3K79 methylation by DOT1L and whether the current cellular functions of DOT1L are mediated through H3K79 methylation only or by the methylation of a wider range of proteins. For example, only a handful of readers of the H3K79 methylation marks have been identified thus far, and whether the different methylation marks have distinct functions in transcriptional regulation and other key functions remains a largely unanswered question. Understanding the molecular mechanisms linking H3K79 methylation to actions within the cell is critical to fully appreciating the contribution of DOT1L and H3K79 methylation to genome function and integrity.

## Figures and Tables

**Table 1:** Transcription elongation complexes relevant to DOT1L function.

Complex	Protein Components	Reference
AEP	AFF1/4, ENL, P-TEFb	[60]
AF4-associated	AFF1, AF9, DOT1L, ENL, MLLT10, P-TEFb	[61]
AF9-associated	AF9, DOT1L (mutually exclusive with AF4/AFF4, AF9, P-TEFb)	[62]
DotCom	AF9, CTNNB1, DOT1L, ENL, MLLT6, MLLT10, SKP1, TRRAP	[63]
EAP	AFF1/3/4, BCOR, CBX8, DOT1L, ENL, P-TEFb, RING1	[64]
EAP core	AFF1, DOT1L, ENL, P-TEFb	[65]
SEC	AFF1/4, AF9, EAF1/2, ELL1/2/3, ENL, P-TEFb	[66]

AEP: AF4, ENL and P-TEFb complex; DotCom: Dot1-containing complex; EAP: ENL-associated proteins complex; SEC: super-elongation complex.
